# An American patient with polyposis carrying a Scandinavian *AXIN2* pathogenic variant

**DOI:** 10.1186/s13053-020-00149-8

**Published:** 2020-07-30

**Authors:** Sarah K. Macklin-Mantia, Douglas L. Riegert-Johnson

**Affiliations:** 1 Department of Clinical Genomics, Mayo Clinic, 4500 San Pablo Road South, Jacksonville, FL 32224 USA; 2 Department of Gastroenterology, Mayo Clinic, 4500 San Pablo Road South, Jacksonville, FL 32224 USA

**Keywords:** *AXIN2*, Hereditary cancer syndrome, Hereditary polyposis, Cancer, Hypodontia

To the Editor,

We report the case of an *AXIN2* pathogenic variant carrier. AXIN2 is a component of the beta catenin destruction complex of the canonical Wnt signaling pathway. *AXIN2* pathogenic variants decrease beta catenin destruction and increase Wnt signaling. APC is one of the other components and germline pathogenic variants in *APC* are associated with familial adenomatous polyposis [1].

*AXIN2* pathogenic variants are associated with the absence of permanent teeth (hypodontia), sparse hair and eye brows (ectodermal dysplasia), and gastrointestinal polyps and cancer. Inheritance is autosomal dominant with variable penetrance. About 30 other cases have been reported since the first cases 17 years ago [2–6]. Given the rarity of pathogenic variants in *AXIN2,* each additional case report is helpful in clarifying the phenotype. This clinical report is noteworthy for being the first of a Scandinavian pathogenic variant carrier outside of Scandinavia and the first report of colonoscopy findings for the *AXIN2* c.1994dupG pathogenic variant.

The patient was a 65 year-old white man referred by his local gastroenterologist in January of 2020. His past medical history included prostate adenocarcinoma (62 years of age, treated with radiation therapy), skin cancer (60 years of age, type unknown), rheumatoid arthritis, glaucoma, hypertension, and obstructive sleep apnea.

On physical examination, he had no features of ectodermal dysplasia and 25 teeth. (Normal adult number of teeth is 32). The patient had had several teeth removed due to decay, and he may have also had absence of permanent teeth. The ongoing COVID-19 pandemic has prevented the patient from attending an expert dental consultation for further evaluation.

The patient has had 57 adenomatous colon polyps removed during four colonoscopies (June 2019, August 2019, November 2019, and March 2020). An upper endoscopy in March of 2020 showed no polyps. Strategies for management of his colon polyps were presented to the patient. For surgery, we offered laparoscopic subtotal colectomy with follow up flexible sigmoidoscopy every six to twelve months. For colonoscopy, we recommended a procedure every six to twelve months based on the number of polyps found. For the present, the patient has chosen to continue with colonoscopy.

**Fig. 1:**
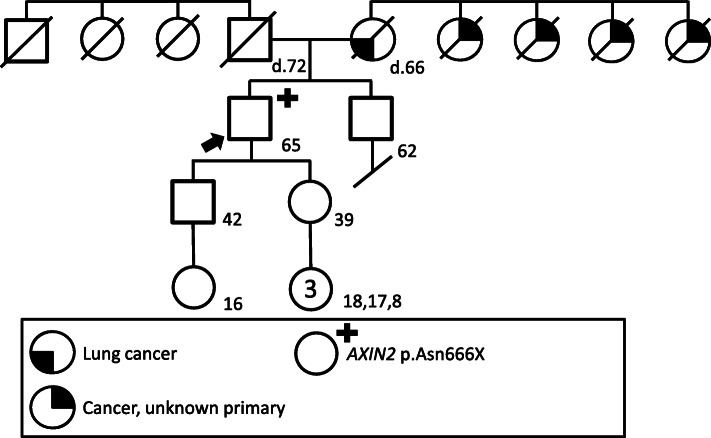
Pedigree. The arrow points to the proband carrying the AXIN2 c.1994dupG pathogenic variant. Current ages or age at death are noted to the right of pedigree symbols.

The patient had limited information on his ancestry and family history; what is known is written below or shown in Fig. 1. He knew of no connection to Scandinavia. He was born in a small town in southeast Georgia, USA with a current population under 15,000 individuals. His mother and father were born in separate, smaller, nearby towns in Georgia with populations less than 5,000 individuals. His mother died at 66 years of age from lung cancer, and his father died at 72 years of age from complications of hypertension. No other family members have had *AXIN2* genetic testing, colonoscopy, or are known to have dental abnormalities. The patient’s family was counseled on their risk and offered cascade testing. With the ongoing COVID-19 pandemic, they do not feel it is safe to travel from their rural home to Mayo Clinic Florida for testing.

Germline genetic testing was ordered through a commercial genetic testing laboratory and included analysis of 70 genes (Ambry genetics – Aliso Viejo, California – CancerNext Expanded with *MSH3*, *NTHL1*, and *AXIN2* added)*.* A pathogenic variant was detected in *AXIN2,* c.1994dupG, located in exon 7, which causes a translational frameshift with a predicted alternate stop codon (p.N666Qfs*41) (NM_004655.3) (ClinVar ID: 536512).

The c.1994dupG pathogenic variant found is the same identified in the initial 2004 Finnish *AXIN2* publication as a de novo case by Lammi and others. The variant was also reported in 2011 by Bergendal and others from Sweden. All reported c.1994dupG cases had oligodontia, none had ectodermal dysplasia, and none had had a colonoscopy. The genome aggregation database (GnomAD) was accessed April 3, 2020 and searched for data on the c.1994dup G variant (17–63,532,584-G-GC (GRCh37). The allele frequency was 0.0006954 (17 heterozygotes). Interestingly, three heterozygotes were in the African population, the others were European (non-Finnish) (12), Finnish (1), and Ashkenazi Jewish (1).

The identification of an American carrier of this pathogenic *AXIN2* variant previously found in Scandinavia raises the possibility of an unidentified, large, American *AXIN2* pathogenic variant family. The same variant reported here has also been reported into ClinVar by another American laboratory. Single nucleotide insertions, such as 1994dupG, are relatively uncommon de novo events and unlikely to be recurrent. The *AXIN2* 1994dupG variant likely follows the common motif in hereditary cancer syndromes of the identification of seemingly unrelated families in the United States with the same pathogenic variant eventually shown to have common descent to a European ancestor (e.g. *EPCAM* pathogenic variants [7].

The small number of known cases with germline *AXIN2* pathogenic variants makes it challenging to accurately assess risks for these individuals. We would ask that any clinicians treating patients with *AXIN2* pathogenic variants contact us. Our group, working with genetic testing laboratories and clinicians, is building an *AXIN2* patient registry.
